# Portal Venous Tumor Thrombosis and Visceral Organ Metastasis without Skeletal Involvement in mCRPC: Adverse Prognostic Indicators on Dual Tracer PET/CT and Clinical Outcome after ^177^ Lu-PSMA-617 PRLT and Cabazitaxel Therapy

**DOI:** 10.1055/s-0044-1788736

**Published:** 2024-07-24

**Authors:** Yeshwanth Edamadaka, Rahul V. Parghane, Sandip Basu

**Affiliations:** 1 Radiation Medicine Centre, Bhabha Atomic Research Centre, Tata Memorial Hospital Annexe, Jerbai Wadia Road, Parel, Mumbai, India; 2 Homi Bhabha National Institute, Mumbai, India

**Keywords:** prostate cancer, metastatic castration-resistant prostate cancer, liver metastasis, portal venous tumor thrombosis, ^68^ Ga-PSMA-11 PET/CT, ^18^ F-FDG-PET/CT, ^177^ Lu-PSMA-617 radioligand therapy

## Abstract

Prostate cancer involving visceral organs are occurrences in the later disease course, usually following regional nodal and skeletal involvement, and are refractory to conventional treatment. A 61-year-old male patient presented with locally advanced disease at presentation, which progressed on androgen deprivation therapy and systemic therapy with involvement of the visceral organs (lungs and liver). Portal venous tumor thrombosis involving the right and main branch was also observed on contrast-enhanced computed tomography (CECT) and magnetic resonance imaging (MRI), which showed intense uptake on ^68^ Ga-labeled prostate-specific membrane antigen positron emission tomography/computed tomography ( ^68^ Ga-PSMA-11 PET/CT) and ^18^ F-fluorodeoxyglucose PET/CT ( ^18^ F-FDG-PET/CT). Post- ^177^ Lu-PSMA-617 radioligand therapy (PRLT) showed mixed response on tumor marker and imaging analysis with survival of 6 months after ^177^ Lu-PSMA radioligand therapy. The high Gleason score, visceral organ metastasis, and increased metabolic activity on FDG were the adverse prognostic factors in the described patient.

## Introduction

Prostate cancer metastasis is often observed in a recognizable pattern involving regional lymph nodes followed by the bones. The visceral organ metastasis usually occurs late in the disease course and is often refractory to conventional treatment. The detection of visceral metastasis has been described to influence the overall survival (OS) and is an important negative prognostic indicator. 1 However, the present understanding of what drives the development of different metastatic patterns in metastatic castration-resistant prostate cancer (mCRPC) is limited. In the presented case, we report an uncommon metastatic spread of castration-resistant prostate cancer (CRPC) with predominant involvement of the visceral organs including lung and liver with portal vein thrombosis detected on dual tracer positron emission tomography (PET)/computed tomography (CT) imaging.

## Case History

**Fig. 1:**
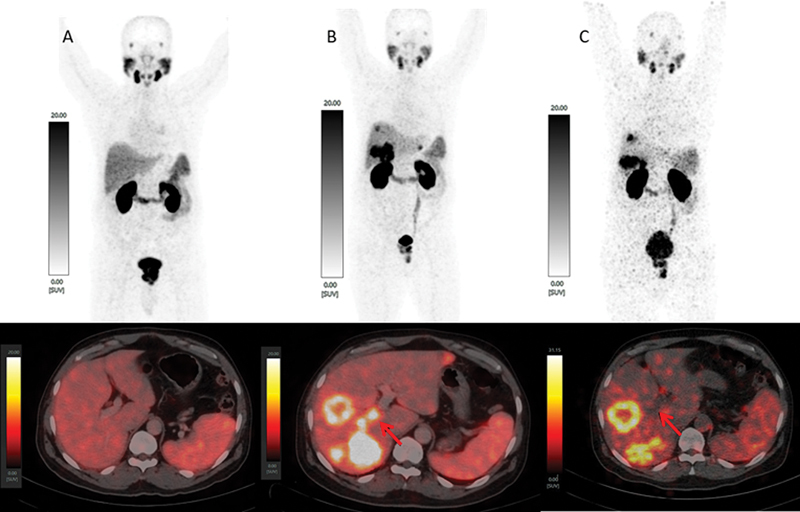
^68^ Ga-labeled prostate-specific membrane antigen positron emission tomography/computed tomography ( ^68^ Ga-PSMA-11 PET/CT) scan. ( **A** ) Maximum intensity projection (MIP) at baseline showed locally advance prostatic carcinoma (upper row) with no visceral organ involvement (lower row fusion image). ( **B** ) MIP showed multiple liver metastasis (upper) and intense ^68^ Ga-PSMA expression in metastatic liver lesion along with right and main portal vein thrombosis (lower fusion image). ( **C** ) MIP showed overall stable disease (upper) and resolution of portal vein thrombosis ( *red arrow* ) and increase in segment V liver metastasis (lower fusion image).

**Fig. 2:**
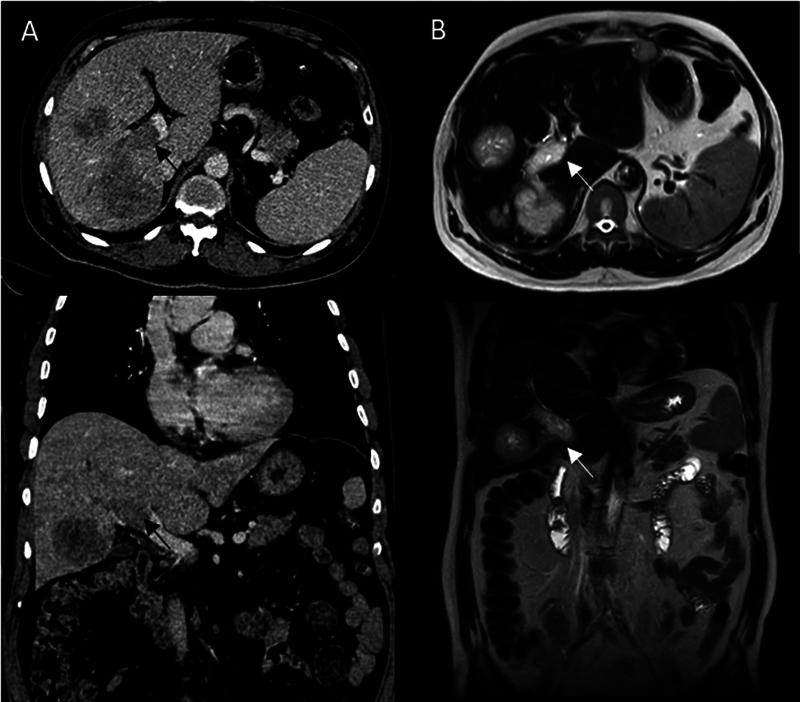
( **A** ) Axial (upper) and coronal (lower) contrast-enhanced computed tomography (CECT) image showing metastatic liver lesions and filling defect in the main portal vein ( *black arrows* ). ( **B** ) Axial (upper) and coronal (lower) T2-weighted magnetic resonance imaging (MRI) showing hyperintense metastatic liver lesion and main portal vein suggesting portal vein thrombosis ( *white arrows* ).

**Fig. 3:**
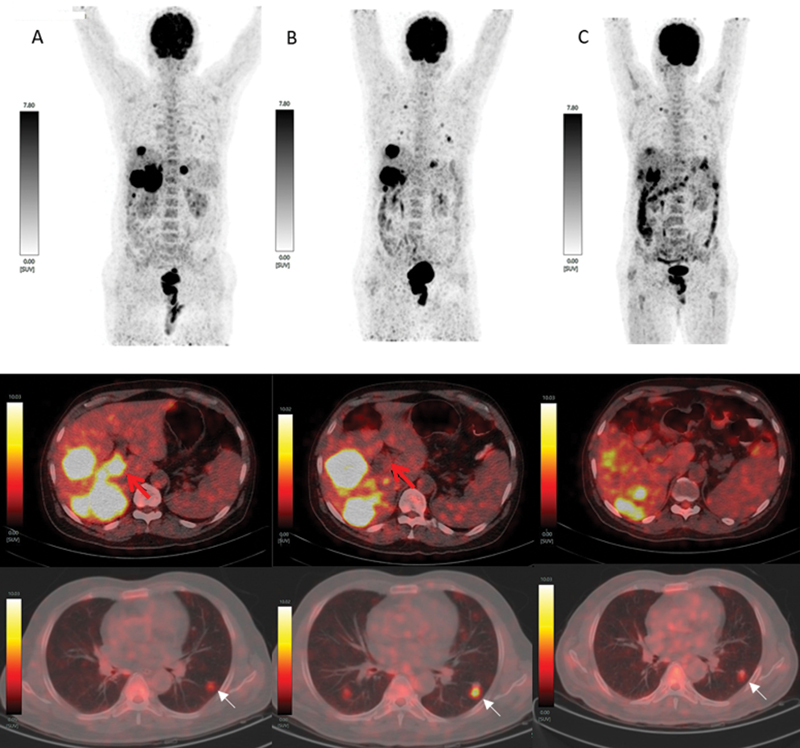
^18^ F-fluorodeoxyglucose positron emission tomography/computed tomography ( ^18^ F-FDG-PET/CT) scan. ( **A** ) Maximum intensity projection (MIP) showing hypermetabolic bulky liver metastasis (upper row), intense ^18^ F-FDG uptake ( *red arrow* ) in tumor thrombosis (middle fusion image) and faint uptake in the left lung nodule (lower fusion image). The scan was done before PSMA-617 radioligand therapy (PRLT) initiation. ( **B** ) MIP showed increased number of lung nodules and increased segment V liver lesions (upper row), resolution of FDG uptake in portal vein thrombosis ( *black arrow* ) and persistent metabolic activity in hepatic lesions (middle fusion image) and increased metabolic activity ( *white arrow* ) in the left and new right lung nodule (lower fusion image). The scan done before the second cycle of PRLT. ( **C** ) MIP showing reduction of metabolic activity in lung and liver lesions (upper row) and decreased metabolic activity in liver and lung lesions (middle and lower fusion image). The scan done before the third cycle of PRLT.

**Fig. 4:**
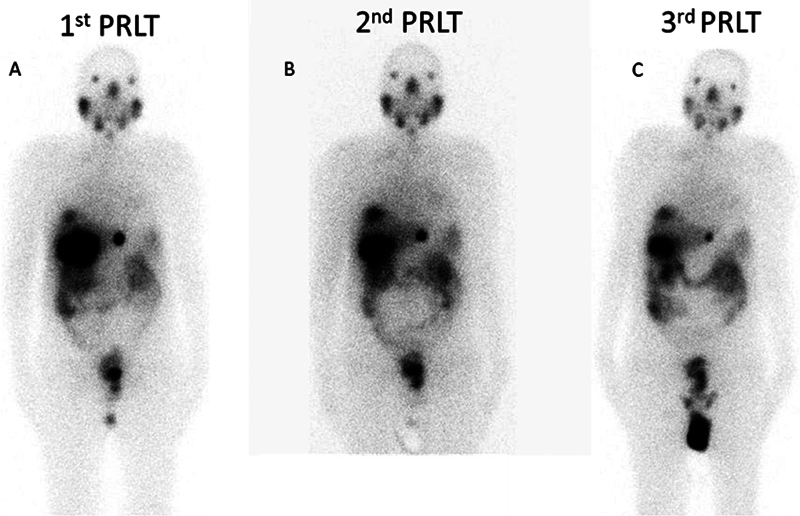
( **A–C** ) Posttherapy ^177^ Lu-prostate-specific membrane antigen radioligand therapy ( ^177^ Lu-PSMA-617 RLT) whole-body (WB) planar images done 24 hours after therapeutic administration showing significant tracer localization in liver metastasis following all three PSMA-617 radioligand therapy (PRLT) cycles.

A 61-year-old man initially presented with lower urinary tract symptoms with serum prostate specific antigen (Sr PSA) of 16.7 ng/mL and sonographic evaluation showing enlarged prostate with heterogenous parenchyma. Prostate biopsy revealed a grade V conventional prostatic adenocarcinoma with maximum Gleason score (GS) of 5 + 5 = 10. An initial ^68^ Ga-prostate-specific membrane antigen ( ^68^ Ga-PSMA-11) PET/CT done for primary staging in view of high-risk disease showed intense uptake in advanced local disease involving the urinary bladder ( Fig. 1 ). As the disease was not amenable to surgery, the patient was planned for antiandrogen (AA) therapy and subsequently systemic hormonal therapy with tablet abiraterone. He presented with hematuria 3 months after initiation of systemic therapy though he showed a significant biochemical response Sr PSA of 0.7 ng/mL. He was planned for palliative radiotherapy (RT) targeting the prostate with a fractionated dose regimen, while he continued abiraterone. He had a biochemical progression (Sr PSA of 15.9 ng/mL) 3 months after palliative RT, which was further evaluated with ^68^ Ga-PSMA-11 PET/CT, which showed disease progression with involvement of the visceral organs including multiple lung nodules and bulky liver lesions ( Fig. 1 ). Intense ^68^ Ga-PSMA-11 uptake was noted in suspicious tumor thrombus in the right and main portal vein, which was further evaluated with triphasic contrast-enhanced CT and magnetic resonance imaging (MRI) showing a filling defect in the venous phase and intermediate to hyperintensity in T2-weighted images, respectively ( Fig. 2 ). We performed ^18^ F-FDG-PET/CT ( Fig. 3 ) to prognosticate and considering an unusual pattern of metastasis that showed intense metabolic activity in primary and metastatic lesions also including the tumor thrombus. The patient was planned for ^177^ Lu-PSMA-617 radioligand therapy (PRLT) 15 months after initial diagnosis. He tolerated PRLT well, but on 2 months of follow-up, we noticed a biochemical progression with Sr PSA almost doubled (Sr PSA 28.7 of ng/mL). The dual tracer PET imaging ( Figs. 1 2 3 ) showed new metastatic lung nodules and increase in the size of liver metastasis but significant resolution in tumor thrombus. The overall finding was suggestive of disease progression. The patient was administered the second cycle of PRLT as a salvage treatment along with cabazitaxel chemotherapy subsequently. Prior to his third cycle of PRLT, he showed stable Sr PSA levels of 26.1 ng/mL, ^18^ F-FDG-PET/CT ( Fig. 3 ) showed decreased metabolic activity in bulky liver lesions, and metastatic lung nodules suggesting favorable response. Posttherapy whole-body (WB) planar scans ( Fig. 4 ) showed significant tracer uptake in prostatic primary and bulky liver lesions, with no discernible uptake in lung nodules. The patient continued their planned chemotherapy post-PRLT, during which he developed increased fatigue with declining overall performance status. The patient succumbed to the disease 6 months after the start of PRLT and 21 months from the initial diagnosis.

## Discussion

Androgen deprivation therapy (ADT) is the first line of treatment for either recurrent or metastatic prostatic cancer. Over the course of treatment, resistance to AA therapies sets in, leading to disease progression termed as CRPC. The responsiveness and overall success of AA therapies is dependent on the site of metastasis. Liver metastasis are described to be associated with poor outcome. 2 Liver metastasis in prostate cancer is usually a later-stage event and, hence, not routinely screened during patient workup. The recent reports found an increase in annual incidence rate of 12.3% of visceral metastasis in prostate cancer patients owing to new survival prolonging therapies. 3 The liver is a challenging metastatic site to study due to its metastatic dormancy with no notable symptoms initially, usually detected when there is widespread liver involvement unlike bone metastasis, which can present with pain. Iwamoto et al reported that prior use of second-generation AA therapies was associated with liver metastasis in mCRPC patients. 4 A postulation is that the liver is responsible for AA metabolism, which may result in liver damaging intermediates initiating an inflammatory and fibrotic response, which may further provide a niche for tumor cell seeding and growth. 5 Vinjamoori et al 6 retrospectively reviewed the atypical metastatic pattern in a cohort of 620 patients of prostatic cancer and concluded that atypical sites were associated with a high GS and that there was no significant correlation with concurrent osseous involvement. Pouessel et al 7 reported that liver metastasis occurred late in the course of the disease with median time from first metastasis to the liver of 17 months and median OS of 6 months. The reported median OS from a meta-analysis of phase III trials treated with docetaxel including 752 men with liver metastasis was 13.5 months compared to 19.4 months for 791 men with lung metastasis. 8

Identification of patients who are most suited for PRLT therapy is critical for outcome as the rate of nonresponders is almost half in the VISION trial due to aggressive tumor biology. 9 The pretherapeutic dual tracer PET/CT can help investigate any mismatch lesions, further impacting treatment selection. PSMA expression would reduce in mCRPC lesions, reducing the sensitivity of ^68^ Ga-PSMA-PET/CT and ^18^ F-FDG-PET/CT can complement evaluation of the tumor heterogeneity in advanced prostate cancer. Buteau et al 10 demonstrated the prognostic role of metabolic tumor burden regardless of treatment assigned requiring treatment intensification, while studies investigating the potential use of the dual tracer PET/CT imaging approach for treatment response are lacking.

The available literature for response and outcome of liver metastases to PRLT in mCRPC patients are lacking. Khreish et al 11 retrospectively studied 28 patients of liver metastasis who received PRLT. In their study, 46% of the patients had hepatic disease control, with complete response and partial response in 21% each. The median progression-free survival (PFS) was 5.7 months and OS was 11.7 months. Patients with hepatic disease control did not reach the median OS in the follow-up period compared to those with progressive hepatic disease with an OS of 6.4 months. Our case presented initially as a locally advanced disease not amenable for surgical resection, which progressed on ADT and systemic therapy with involvement of the visceral organs including the lungs and the liver; the visceral metastasis was diagnosed 15 months from the initial diagnosis. After the first cycle of PRLT, we found a biochemical and image-wise progression with an OS of 6 months post-PRLT, similar to that reported in the literature. This is similar to the aggressive cohort of patients described by Pouessel et al 7 and Khreish et al 11 reporting a median OS of 6.4 months in patients with progressive hepatic disease. Recent studies showed improved clinical outcome using alpha therapy ( ^225^ Ac-PSMA-617) in patients in whom ^177^ Lu therapy has failed. 12 Feuerecker et al 13 reported good antitumor effect using alpha therapy in cases resistant to ^177^ Lu-PSMA-617. Interestingly, however, liver metastasis showed only modest improvement in serum PSA and clinical-based PFS with grade 3/4 hematological toxicity reported in one-third of patients.

## Conclusion

Prostatic cancer metastasis to visceral organs before skeletal or nodal disease is unusual, but the clinical outcome in comparison to later onset was similar. High GS, visceral organ metastasis, and increased metabolic activity were the negative prognostic factors in the described patient. Biochemical progression after PRLT can predict the treatment outcome, and the potential for combination treatment needs to be explored in this subgroup of patients.
